# Atrial tachyarrhythmia prevention by Shensong Yangxin after catheter ablation for persistent atrial fibrillation: the SS-AFRF trial

**DOI:** 10.1093/eurheartj/ehae532

**Published:** 2024-08-23

**Authors:** He Huang, Yu Liu, Wei Shuai, Chenyang Jiang, Menghe Zhang, Xiufen Qu, Wenqing Zheng, Hao Yang, Fan Liu, Bo Yu, Manhua Chen, Bin Mu, Chen Yao, Yanhong Tang, Congxin Huang, Feifan Ouyang, Zhenhua Jia

**Affiliations:** Department of Cardiology, Renmin Hospital of Wuhan University, 238 Jiefang Road, Wuhan 430060, China; Cardiovascular Research Institute of Wuhan University, 238 Jiefang Road, Wuhan 430060, China; Hubei Key Laboratory of Cardiology, 238 Jiefang Road, Wuhan 430060, China; Department of Cardiology, Renmin Hospital of Wuhan University, 238 Jiefang Road, Wuhan 430060, China; Cardiovascular Research Institute of Wuhan University, 238 Jiefang Road, Wuhan 430060, China; Hubei Key Laboratory of Cardiology, 238 Jiefang Road, Wuhan 430060, China; Department of Cardiology, Renmin Hospital of Wuhan University, 238 Jiefang Road, Wuhan 430060, China; Cardiovascular Research Institute of Wuhan University, 238 Jiefang Road, Wuhan 430060, China; Hubei Key Laboratory of Cardiology, 238 Jiefang Road, Wuhan 430060, China; Department of Cardiology, Sir Run Run Shaw Hospital, Zhejiang University School of Medicine, Hangzhou, China; Department of Cardiology, The Second Affiliated Hospital of Shandong University of Traditional Chinese Medicine, Jinan, China; Department of Cardiology, The First Affiliated Hospital of Harbin Medical University, Harbin, China; Department of Cardiology, Weihai Central Hospital, Weihai, China; Department of Cardiology, The First Affiliated Hospital of Wannan Medical College, Wuhu, China; Department of Cardiology, The Second Hospital of Hebei Medical University, Shijiazhuang, China; Department of Cardiology, The First Hospital of China Medical University, Shenyang, China; Department of Cardiology, The Central Hospital of Wuhan, Tongji Medical College, Huazhong University of Science and Technology, Wuhan, China; Department of Cardiology, General Hospital of Ningxia Medical University, Yinchuan, China; Peking University Clinical Research Institute, Peking University First Hospital, Beijing, China; Department of Cardiology, Renmin Hospital of Wuhan University, 238 Jiefang Road, Wuhan 430060, China; Cardiovascular Research Institute of Wuhan University, 238 Jiefang Road, Wuhan 430060, China; Hubei Key Laboratory of Cardiology, 238 Jiefang Road, Wuhan 430060, China; Department of Cardiology, Renmin Hospital of Wuhan University, 238 Jiefang Road, Wuhan 430060, China; Cardiovascular Research Institute of Wuhan University, 238 Jiefang Road, Wuhan 430060, China; Hubei Key Laboratory of Cardiology, 238 Jiefang Road, Wuhan 430060, China; Hongkong Asia Medical Group/University Medical Center Hamburg-Eppendorf, University Heart and Vessel Center Hamburg, Martinistraße 52, Hamburg 20246, Germany; Department of Cardiology, Hebei Yiling Hospital, 385 Xinshibei Road, Shijiazhuang 050091, China; State Key Laboratory for Innovation and Transformation of Luobing Theory, 238 Tianshan Street, Shijiazhuang 050035, China

**Keywords:** Traditional Chinese medicine, Shensong Yangxin capsule, Persistent atrial fibrillation, Catheter ablation

## Abstract

**Background and Aims:**

Despite advances in technology and techniques, the recurrence rate of persistent atrial fibrillation (AF) following catheter ablation remains high. The Shensong Yangxin (SSYX) capsule, a renowned traditional Chinese medicine formula, is used in the treatment of cardiac arrhythmias. This trial aimed to investigate whether the SSYX can improve clinical outcomes in patients who have undergone catheter ablation for persistent AF.

**Methods:**

A multi-centre, randomized, double-blind, placebo-controlled clinical trial was conducted at 66 centres in China among 920 patients with persistent AF undergoing first ablation. Participants were randomized to oral SSYX, 1.6 g (.4 g/granule) thrice daily (*n* = 460), or matched placebo (*n* = 460) for 12 months. The primary endpoint was recurrent atrial tachyarrhythmias lasting for ≥30 s following a blanking period of 3 months. Secondary endpoints included time to first documented atrial tachyarrhythmias, AF burden, cardioversion, stroke/systemic embolism, changes in echocardiographic parameters, and quality-of-life (QoL) score. Analyses were performed according to the intention-to-treat principle.

**Results:**

A total of 920 patients underwent randomization (460 assigned to SSYX group and 460 assigned to placebo group). During the follow-up of 12 months, patients assigned to SSYX had a higher event-free rate from recurrent atrial tachyarrhythmias when compared with the placebo group (12-month Kaplan–Meier event-free rate estimates, 85.5% and 77.7%, respectively; hazard ratio, .6; 95% confidence interval .4–.8; *P* = .001). Patients assigned to receive SSYX had a better QoL score at 12 months compared to those randomized to placebo. There was no significant difference in the incidence of serious adverse events between the two groups.

**Conclusions:**

Treatment with SSYX following radiofrequency catheter ablation for persistent AF reduced the incidence of recurrent atrial tachyarrhythmias and led to clinically significant improvements in QoL during a 12-month follow-up in a Chinese population.

## Structured Graphical Abstract

**Structured Graphical Abstract:**
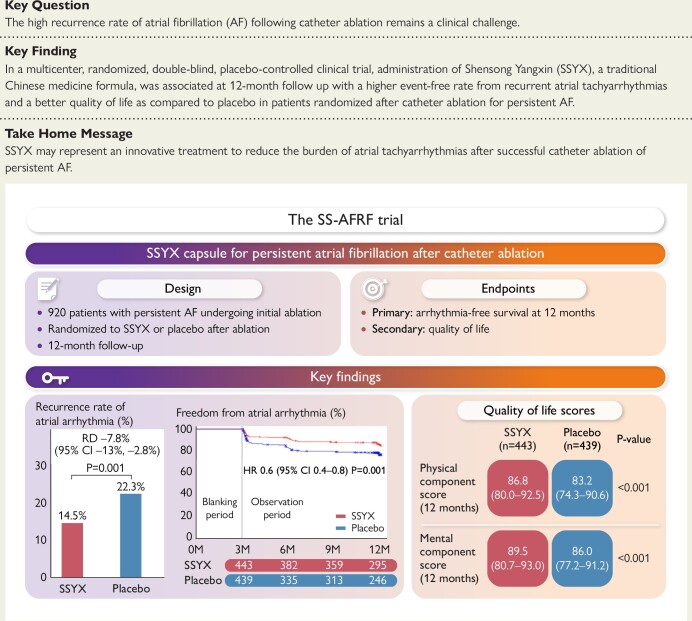
A total of 920 Chinese patients with persistent AF undergoing first ablation were randomized to oral SSYX or matched placebo for 12 months. The recurrence of atrial arrhythmia (left), Kaplan–Meier curves depicting the time to the first recurrence of atrial arrhythmia after the 3-month blanking period (middle), and quality of life at 12-month follow-up (right) were evaluated. SSYX, Shensong Yangxin; CI, confidence interval; AF, atrial fibrillation; RD, rate difference; HR, hazard ratio.

**See the editorial comment for this article ‘Traditional Chinese medicine: cardiovascular drug development through a holistic framework’, by M. Packer, https://doi.org/10.1093/eurheartj/ehae625.**

## Introduction

The burden of atrial fibrillation (AF) is increasing in prevalence and healthcare costs in China and worldwide.^1^ Epidemiological evidence indicates that the incidence of AF among Chinese adults stands at 1.6% for the general population, 1.7% for men, and 1.4% for women.^2^ It can substantially impair quality of life (QoL) and increase the risks of stroke, heart failure, and mortality.^3–5^ Rhythm control forms the cornerstone of AF integrated management and is associated with reduced adverse cardiovascular outcomes.^6^ Despite advances in technology and techniques, the recurrence rate of persistent AF following catheter ablation remains high.^7–9^ Short-term use of anti-arrhythmic drugs (AADs) following ablation can significantly reduce the risk of early atrial arrhythmia recurrence, yet it does not correspondingly lower the risk of late atrial arrhythmia recurrence.^10,11^ While extended use of AADs beyond the post-ablation blanking period may decrease the recurrence of atrial tachyarrhythmias, the high rate of medication discontinuation indicates that side effects or inefficacy might limit the long-term use of AADs after ablation.^12,13^

Traditional Chinese medicine boasts a history spanning over 2000 years and is extensively utilized in clinical settings. The Shensong Yangxin (SSYX) capsule, a renowned traditional Chinese patent medicine, comprises 12 herbal materials. It was approved by the State Food and Drug Administration of China in 2003 (File No. Z20030058) and has been reported to be effective in treating cardiac arrhythmia. Preclinical studies have demonstrated that SSYX effectively inhibits myocardial remodelling, reduces cardiomyocyte fibrosis, blocks multiple ion channels, regulates cardiac autonomic nerve function, and alleviates cardiac loads, thus playing a significant anti-arrhythmic role.^14–17^ Randomized clinical trials demonstrated that SSYX significantly reduced the frequency of premature ventricular contractions and paroxysmal AF.^18–21^ More importantly, SSYX exhibits an excellent profile of safety, potentially safer than AADs.^18,21^ Gastrointestinal reactions such as nausea, vomiting, loss of appetite, constipation, and thirst are the most common side effects of SSYX, but they occur infrequently.^22^ However, whether the long-term use of SSYX after catheter ablation for persistent AF can effectively and safely reduce the recurrence of AF is unclear. Hence, we conducted a multi-centre, randomized, double-blind, placebo-controlled clinical trial, evaluating whether oral SSYX following radiofrequency ablation can reduce the recurrence of AF in patients with persistent AF.

## Methods

### Trial design

The trial was designed as a multi-centre, randomized, double-blind, placebo-controlled clinical trial. The trial was conducted in accordance with the Helsinki Declaration and was approved by the institutional ethics committee of each participating hospital. This trial was designed and coordinated by the authors and over-seen by a clinical event committee and a data and safety monitoring board. The trial was registered at http://www.chictr.org.cn/showproj.aspx?proj=44825. Prior to trial start, written informed consent was obtained from all participants prior to inclusion. Enrolment began in November 2019 and was completed in October 2021. Follow-up was finalized in November 2022.

### Trial participants

Patients were recruited from 66 experienced centres in China. Patients with persistent AF undergoing first ablation were eligible for inclusion. Exclusion criteria were age <18 or >75 years, New York Heart Association class IV heart failure, left ventricular ejection fraction <40%, left atrial diameter >50 mm, very long-lasting (≥5 years) AF, presence of left atrial/left atrial appendage thrombosis, severe hepatic and renal insufficiency (alanine aminotransferase ≥3.0 upper limit of normal or estimated glomerular filtration rate <50.0 mL/min/1.73 m^2^), valvular AF, hyperthyroidism, acute coronary syndrome or significant coronary artery disease requiring stent implantation, open cardiac surgery within the past 6 months, pregnant women, intolerance for optimal anticoagulation, inability to be followed at the outpatient clinic for 12 months, and unwillingness to sign the consent form for participation.

### Ablation, randomization, and treatment

All patients underwent pulmonary vein isolation (PVI) by radiofrequency ablation. The electrophysiological endpoint of PVI was bidirectional conduction block between left atrium and pulmonary veins. Additional ablation including linear ablation, continuous fractionated atrial electrogram ablation, and rotor ablation was left to the discretion of the operating electrophysiologists.

Following ablation, patients were randomly assigned to oral SSYX therapy or matched placebo. Shensong Yangxin or placebo (Shijiazhuang Yiling Pharmaceutical Co., Ltd, Hebei, China) was initiated within 48 h after ablation with a dose of 1.6 g (.4 g/granule) thrice daily for 12 months. Use of AADs during the blanking period was allowed, but discontinuation after the blanking period was strongly recommended. Other anti-arrhythmic traditional Chinese medicine or proprietary Chinese medicine was avoided during the whole trial period.

### Follow-up

Patients were recommended to receive oral anticoagulant therapy for at least 2 months. Patients were scheduled to receive periodical follow-up at 3, 6, 9, and 12 months after the initial ablation procedure. The time window for each visit was 91–97 days for 3 months, 173–187 days for 6 months, 263–277 days for 9 months, and 353–367 days for 12 months. Except for the 9-month follow-up, each follow-up comprised a detailed history, a routine physical examination, 24-h Holter monitoring, and laboratory testing, and all patients completed questionnaires regarding QoL using the Short Form 36 Health Survey (SF-36). In addition to the scheduled follow-up, the trial participants had free access to healthcare providers. They were strongly recommended to visit a healthcare provider if they felt symptoms possibly due to an arrhythmia or noticed any irregularity of their peripheral pulse by routine self-measurement. An electrocardiogram (ECG) was performed at every additional visit, and cases with symptoms or findings suggestive of recurrence underwent 24-h Holter monitoring.

### Endpoints

The primary endpoint of the trial was the recurrence of AF following a blanking period of 3 months. Recurrence of AF was defined as the occurrence of confirmed atrial tachyarrhythmia lasting ≥30 s, including AF, atrial flutter (AFL), or atrial tachycardia (AT). An independent events evaluation committee, whose members were unaware of any group allocation, adjudicated whether AF recurred or not. Secondary endpoints included time to first documented atrial tachyarrhythmias, AF burden (cumulative duration of all AF episodes on the 24-h Holter monitoring), incidence of cardioversion, incidence of stroke/systemic embolism, echocardiographic parameters, and QoL score. The assessments of safety and tolerability were based on spontaneous reports of adverse events, vital signs, and laboratory measurements.

### Sample size calculation

The recurrence rate of AF in patients with persistent AF was assumed to be 39.8% at 12 months after catheter ablation (AF/AFL/AT lasting for ≥30 s recorded by ECG 3 months after catheter ablation was considered as AF recurrence).^6^ We estimated that the recurrence rate of AF could be reduced to 30% in patients with AF after radiofrequency catheter ablation treated with the SSYX. Taking *α* = .05 and *β* = .2, using the bilateral test, the number of cases in the two groups was 368. Considering a 20.0% drop-off rate, we finally enrolled 460 participants in each group.

### Statistical analysis

The full statistical analysis plan can be found in the Supplementary data. The primary efficacy analysis was based on the intention-to-treat principle according to the allocated treatment groups. Categorical variables were compared with the *χ*^2^ test or Fisher’s exact test. The relative risk was calculated with 95% confidence intervals (CIs). Continuous variables were compared using the Student’s *t*-test or Wilcoxon rank-sum test based on their distributions. Event-free rate was estimated by the Kaplan–Meier method, and the log-rank test assessed differences. Statistical analysis software is SAS®9.4 software. The sample size calculation software is PASS13. All statistical tests are carried out bilaterally, and the difference between the two tests will be considered statistically significant if the *P*-value is ≤.05.

## Results

### Trial population

**Figure 1:**
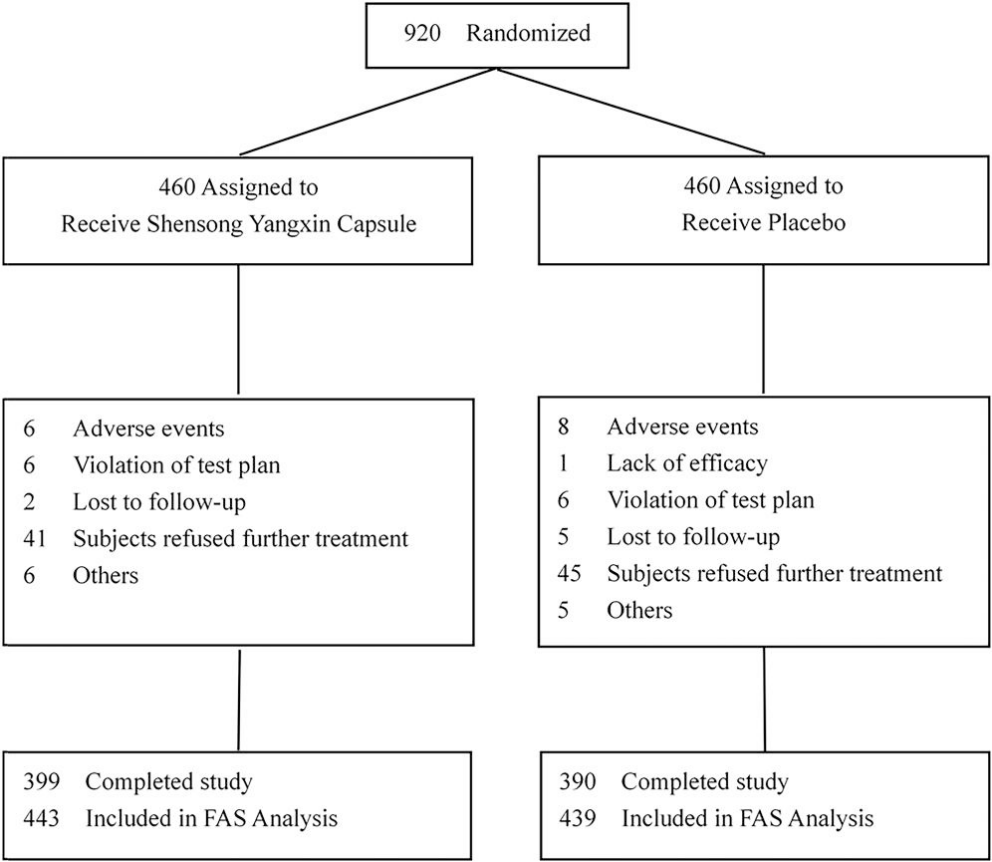
Flow diagram illustrating the number of patients in each group throughout the trial

**Figure 2:**
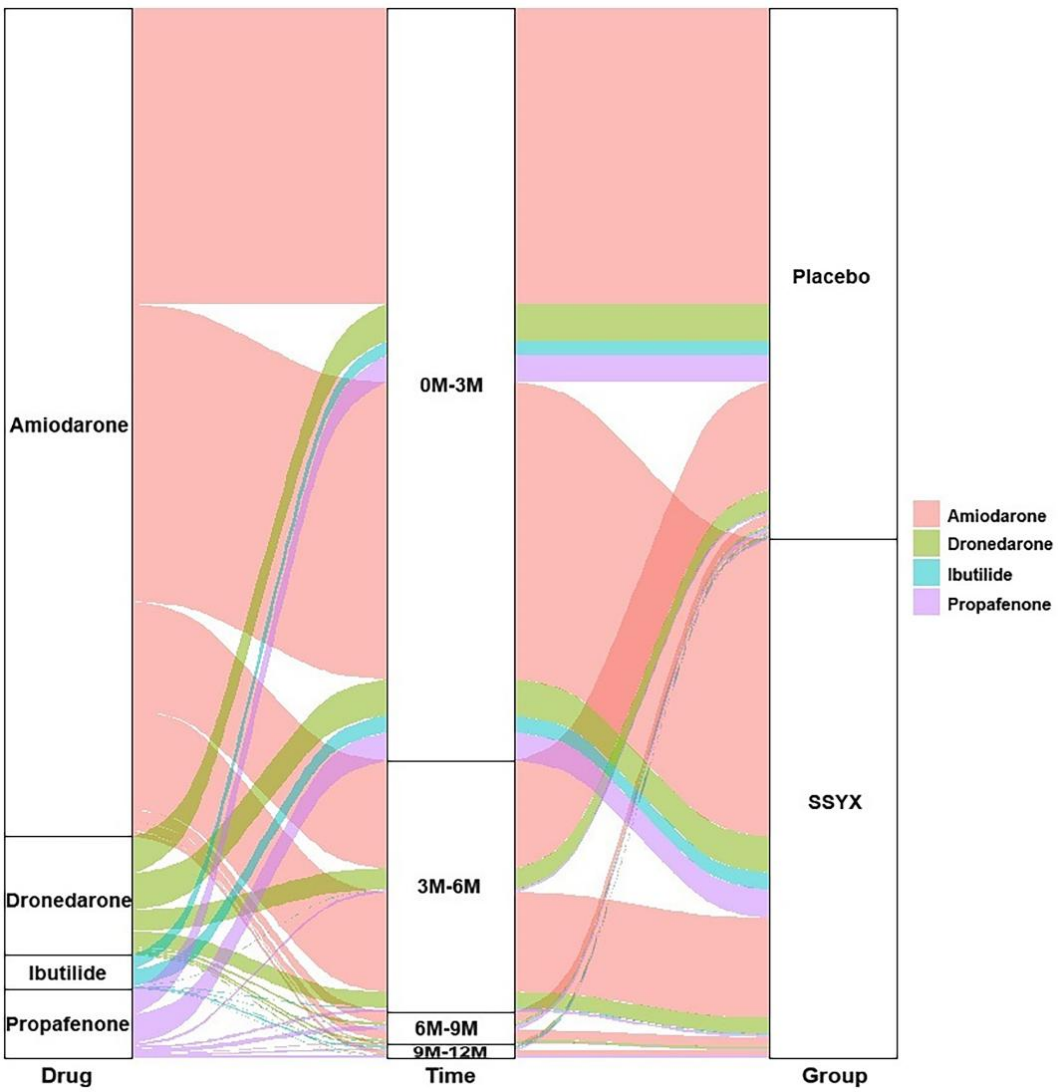
The Sankey diagram displays the proportions of the class I or III anti-arrhythmic drugs used in each group during various follow-up periods after ablation, with thicker branches indicating higher usage rates of the drugs

Between November 2019 and October 2021, a total of 920 patients at 66 centres were enrolled and randomized to the SSYX (*n* = 460) and placebo groups (*n* = 460) during the trial. One hundred and thirty-one patients (61 in SSYX group and 70 in placebo group) discontinued the trial prematurely. An overview of the trial population is shown in *Figure 1*. There were no significant differences in baseline characteristics or ablation procedural characteristics between the two groups, except for sex ratio (*Tables 1* and *2*). Patients were followed for 12 months. During the blanking period, 378 patients (86.1%) in the placebo group and 378 (85.3%) in the SSYX group received an AAD (class I or III) therapy (*P* > .05). However, 68.5% patients during 3–6 months, 96.1% during 6–9 months, and 98.3% during 9–12 months post-ablation discontinued class I or III AADs. The details of the AADs are shown in Supplementary data online, *Table S1*, and *Figure 2*. The most frequently used AAD was amiodarone, followed by dronedarone.

**Table 1:** Baseline characteristics

	SSYX (*n* = 443)	Placebo (*n* = 439)	*P-*value
Age, years, median (IQR)	62 (54, 68)	63 (55, 68)	.411
Men, *n* (%)	342 (77.2)	304 (69.3)	.008
Body mass index, kg/m^2^, median (IQR)	24.9 (22.8, 27.2)	25.3 (23.2, 27.4)	.263
Long-standing persistent AF, *n* (%)	46 (10.4)	54 (12.3)	.369
Previous AADs^a^, *n* (%)	82 (18.5)	88 (20.0)	.563
Left atrial diameter, mm, median (IQR)	42 (39, 45)	42 (38, 45)	.692
Left ventricular ejection fraction, %, median (IQR)	60 (55, 64)	60 (56, 65)	.262
Hypertension, *n* (%)	239 (54.0)	236 (53.8)	.954
Coronary artery disease, *n* (%)	66 (14.9)	67 (15.3)	.880
Previous TIA/stroke, *n* (%)	55 (12.0)	42 (9.6)	.176
Diabetes mellitus, *n* (%)	71 (16.0)	63 (14.4)	.488
History of heart failure, *n* (%)	60 (13.5)	63 (14.4)	.729
CHA_2_DS_2_-VASc score, median (IQR)	2 (1, 3)	2 (1, 3)	.948
CHA_2_DS_2_-VASc score >2, *n* (%)	249 (56.2)	252 (57.4)	.772
Oral anticoagulants at discharge			
Warfarin, *n* (%)	22 (5.0)	12 (2.7)	.085
Dabigatran, *n* (%)	97 (21.9)	88 (20.0)	.500
Xa inhibitor, *n* (%)	310 (70.0)	324 (73.8)	.206

SSYX, Shensong Yangxin; AF, atrial fibrillation; AAD, anti-arrhythmic drug; TIA, transient ischaemic attack; CHA_2_DS_2_-VASc, congestive heart failure, hypertension, age ≥75 years (doubled), diabetes mellitus, prior stroke or transient ischaemic attack (doubled), vascular disease, age 65–74 years, and sex category (female); IQR, interquartile range.

^a^Number of previous attempted class I or class III AADs.

**Table 2:** Ablation procedural characteristics

	SSYX (*n* = 443)	Placebo (*n* = 439)	*P-*value
Pulmonary vein isolation, *n* (%)	443 (100.0)	439 (100.0)	-
Roof line ablation, *n* (%)	259 (58.5)	247 (56.3)	.509
Mitral isthmus linear ablation, *n* (%)	123 (27.8)	121 (27.6)	.946
CFAE ablation, *n* (%)	33 (7.4)	28 (6.4)	.531
Rotor ablation, *n* (%)	3 (.7)	3 (.7)	>.999
Superior vena cava isolation, *n* (%)	21 (4.7)	22 (5.0)	.852
Cavotricuspid isthmus ablation, *n* (%)	68 (15.3)	61 (13.9)	.541
Cardioversion, *n* (%)	290 (65.5)	287 (65.4)	.978

SSYX, Shensong Yangxin; CFAE, complex fractionated atrial electrogram.

### Primary endpoint

**Figure 3:**
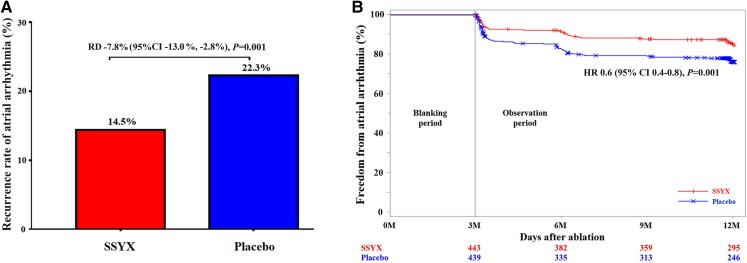
(*A*) Bar chart illustrating differences in the first recurrence of atrial arrhythmia from 3 to 12 months of follow-up after catheter ablation between the placebo and the SSYX groups. (*B*) Kaplan–Meier curves depicting the time to the first recurrence of atrial arrhythmia after the 3-month blanking period in the placebo and the SSYX groups

At 12 months, patients assigned to the SSYX group exhibited significantly higher event-free survival from recurrent atrial tachyarrhythmias compared to those in the placebo group (85.5% vs. 77.7%, *P* = .001) (*Figure 3A*). Time to first documented incidence of AF was plotted using the Kaplan–Meier method (*Figure 3B*).

### Main secondary outcomes

Compared with the placebo group, SSYX significantly reduced the AF burden at 3 and 6 months post-ablation (2.8% vs. 7.6%, *P* = .002 and 3.3% vs. 7.7%, *P* = .025, respectively) as shown in Supplementary data online, *Table S2*. However, no significant difference was observed in AF burden at 12 months post-ablation between the two groups (5.1% vs. 7.9%, *P* = .104). Additionally, the time to first recurrence of AF, rate of electrical cardioversion, incidence of stroke, and echocardiographic parameters were comparable between the SSYX and placebo groups, as detailed in Supplementary data online, *Table S2*.

Regarding QoL, there were significant differences in physical component score (PCS) or mental component score (MCS) between the placebo and SSYX group at 3, 6, and 12 months post-ablation. Both PCS and MCS showed significant improvement over time in the SSYX group, while QoL scores remained stable in the placebo group (*Table 3*).

**Table 3:** Quality of life scores

	SSYX (*n* = 443)	Placebo (*n* = 439)	Estimated difference (95% CI)^a^	*P-*value
Physical component score, median (IQR)				
Baseline	74.3 (62.3, 84.9)	74.3 (61.1, 84.9)	.0 (−1.9, 1.9)	.926
3 months	84.3 (74.3, 90.6)	81.9 (72.5, 89.4)	1.9 (.0, 3.1)	.077
6 months	85.7 (77.4, 92.5)	83.8 (74.3, 90.6)	1.9 (.0, 3.7)	.031
12 months	86.8 (80.0, 92.5)	83.2 (74.3, 90.6)	3.0 (1.9, 4.6)	<.001
Mental component score, median (IQR)				
Baseline	77.2 (59.7, 87.7)	79.0 (59.7, 87.7)	.0 (−1.8, 1.8)	.882
3 months	84.2 (73.7, 91.2)	82.5 (71.9, 91.2)	.0 (−1.8, 1.7)	.400
6 months	86.0 (77.2, 91.2)	84.2 (75.4, 91.2)	1.7 (.0, 3.5)	.056
12 months	89.5 (80.7, 93.0)	86.0 (77.2, 91.2)	3.5 (1.8, 5.2)	<.001

SSYX, Shensong Yangxin; IQR, interquartile range.

^a^Between-group differences are expressed in a pseudo-median difference calculated with the use of the Hodges–Lehmann estimate based on the Mann–Whitney *U* test; because of the use of the Hodges–Lehmann estimator, the estimated difference is not the crude difference between the medians.

### Adverse clinical outcomes

A total of 909 patients (457 in the SSYX group and 452 in the placebo group) were included in the safety set analyses (*Table 4*). During the follow-up, five patients died, one suffered from ischaemic stroke, and six were readmitted to the hospital for heart failure in the SSYX group. In the placebo group, three patients died, three suffered from ischaemic stroke, one patient was readmitted to the hospital for heart failure, and two suffered from intracranial haemorrhage. We found no significant difference in the proportion of patients with serious adverse events between groups (8.5% for SSYX vs. 11.5% for placebo, *P* = .151). The analysis of drug-induced adverse events revealed no significant difference between the two groups. In addition, there was no significant difference in liver and kidney function, and serum lipid levels between the two groups (see Supplementary data online, *Table S3*).

**Table 4:** Adverse clinical outcomes

	SSYX		Placebo		*P-*value
	*n* (%)	*N*	*n* (%)	*N*	
AEs	308 (67.4)	547	303 (67.0)	555	.944
SAEs	39 (8.5)	50	52 (11.5)	63	.151
Death	5 (1.1)	5	3 (.7)	3	.725
Ischaemic stroke/TIA	1 (.2)	1	3 (.7)	3	.371
Intracranial haemorrhage	0 (.0)	0	2 (.4)	2	.247
Myocardial infarction	0 (.0)	0	2 (.4)	2	.247
Hospitalization for heart failure	6 (1.3)	6	1 (.2)	1	.124
Side effects related to study drugs	7 (1.5)	11	13 (2.9)	15	.182

SSYX, Shensong Yangxin; AE, adverse event; SAE, serious adverse event; TIA, transient ischaemic attack; *n*, the number of patients with one or more AEs; *N*, the number of AEs.

## Discussion

This trial is the first randomized, double-blind, placebo-controlled clinical trial to evaluate whether oral SSYX following radiofrequency catheter ablation for persistent AF can reduce the risk of AF recurrence. We found that SSYX significantly reduced the recurrence of atrial tachyarrhythmias in the first year after radiofrequency catheter ablation. In addition, SSYX treatment led to clinical improvements in QoL (*Structured Graphical Abstract*).

Catheter ablation is a well-established therapy for rhythm control in AF management. However, despite time-consuming efforts and the latest ablation technology, clinical outcomes of catheter ablation for AF remain unsatisfactory because of the high recurrence rate, especially for persistent AF. In daily practice and ablation trials, AADs are commonly prescribed to prevent AF recurrence. In the 5A Study, 110 patients undergoing catheter ablation for paroxysmal AF were randomly assigned to a 6-week administration of AADs or no treatment. Despite a significant reduction in arrhythmia recurrence within 6 weeks, there was no difference in the arrhythmia-free rates at the 6-month follow-up.^23^ In the AMIO-CAT trial, 212 patients undergoing catheter ablation for paroxysmal or persistent AF were randomly assigned to 8-week use of amiodarone or placebo. Although amiodarone significantly prolonged the time to first recurrence during a 3-month blanking period, it did not substantially reduce the incidence of recurrent atrial tachyarrhythmias at 6 months.^7^ Also, in the EAST-AF trial, 2038 patients who had undergone radiofrequency catheter ablation for paroxysmal, persistent, or long-lasting AF were randomized to either 90-day use of class I or III AADs or control. Consistent with the results of the AMIO-CAT trial, the short-term effect of AADs was observed, but discontinuation of the drug led to similar arrhythmia-free rates at 1 year.^10^ In the POWDER-AF trial, 153 patients free of paroxysmal AF at the end of 3 months of post-ablation blanking period were randomized to continue previously ineffective AAD therapy or discontinue AAD therapy. The results showed that continued use of previously ineffective AAD therapy significantly reduced the recurrence of atrial tachyarrhythmia in the first year post-ablation.^11^ A multi-centre observational registry including 144 consecutive patients with drug-resistant AF (64% with paroxysmal AF) who underwent a first catheter ablation reported that maintaining AAD therapy after the blanking period was associated with reducing AF recurrence over a median 5-year follow-up.^24^ These findings suggest that combining catheter ablation and AAD therapy may be an effective treatment strategy for improving clinical outcomes of catheter ablation for AF. However, the results from the POWDER-AF2 trial with 200 patients showed that there was no benefit in continuing previously ineffective AAD therapy beyond the blanking period after catheter ablation in patients with persistent AF.^25^ In addition, although long-term use of AADs after AF ablation may be effective in reducing the risk of arrhythmia recurrence in some patients, the beneficial anti-arrhythmic effects of AADs may be offset by their side effects, especially in elderly patients or those with structural heart disease and/or heart failure.^26^ Therefore, in real life, AADs are often discontinued beyond the blanking period. Noseworthy *et al*.^12^ observed that initiation of an AAD at discharge of catheter ablation is associated with a significant reduction in readmission within 90 days. However, AADs were discontinued in 44.5% of patients at 3 months. Consistent with previous studies, our results showed that the majority of patients discontinued class I or III AADs beyond the blanking period. A high rate of medication discontinuation suggests that side effects or inefficacy may limit long-term AAD use post-ablation.

This trial provides direct proof that long-term use of SSYX post-ablation is effective and safe in reducing AF recurrence in patients with persistent AF. Several previous studies demonstrated the anti-arrhythmic effects of SSYX. Zou *et al*.^21^ reported that SSYX had significant therapeutic efficacy in reducing premature ventricular contraction numbers and alleviating premature ventricular contraction-related symptoms compared with placebo or mexiletine. For congestive heart failure patients with frequent premature ventricular contractions, SSYX was demonstrated to have the benefits of premature ventricular contraction suppression and cardiac function improvement with good compliance on a background of standard treatment for congestive heart failure.^20^ A randomized, double-blind and controlled multi-centre trial showed that SSYX and propafenone have comparable efficacy in the treatment of paroxysmal AF, and SSYX has an excellent profile of safety.^18^ A Bayesian network meta-analysis study showed that AADs combined with traditional Chinese formulas, including SSYX, had higher efficacy and lower toxicity than other treatment alternatives for paroxysmal AF.^22^ Our trial, for the first time, revealed that the SSYX reduced the recurrence of AF in patients with persistent AF after radiofrequency catheter ablation, and no serious adverse event was observed after the 12-month treatment.

The underlying mechanisms for the anti-arrhythmic effects of SSYX remain to be elucidated. Shensong Yangxin is composed of 12 different ingredients: (i) the Panax ginseng C. A. Mey. (Ren-Shen); (ii) *Tuber of dwarf lilyturf.* (Mai-Dong); (iii) *Taxillus chinensis* Danser (Sang-Ji-Sheng); (iv) *Cornus officinalis* Sieb. et Zucc. (Shan-zhu-Yu); (v) *Coptis chinensis* Franch. (Huang-Lian); (vi) *Salvia miltiorrhiza* Bge. (Dan-Shen); (vii) *Semen ziziphi spinosae* (Suan-Zao-Ren); (viii) *Fructus Schisandrae Chinensis* (Wu-Wei-Zi); (ix) *Radix Paeoniae Rubra* (Chi-Shao); (x) *Eupolyphaga sinensis* Walker (Tu-Bie-Chong); (xi) sternum/breastbone of a bird (Long-Gu); and (xii) *Radix et Rhizoma Nardostachyos* (Gan-Song). These ingredients are combined in capsules that are available for prescription. It is a new anti-arrhythmia drug developed under the guidance of the theory of venation, with ‘improving qi and nourishing Yin, activating blood and collaterals, clearing the heart and calming the spirit’ as the treatment methods and ‘temperature, clarity, communication and tonic’ as the prescription principles. Shensong Yangxin could affect cardiac action potentials by regulating many ion channels, such as L-type calcium channel current, Na channel current, inward rectified potassium current, and delayed rectified current, thus exerting a broad-spectrum anti-arrhythmia effect.^16,27,28^ Meanwhile, SSYX has a non-ion channel regulating effect. Shensong Yangxin was reported to reduce AF susceptibility by inhibiting atrial fibrosis in rats with post-myocardial infarction heart failure.^29^ Shensong Yangxin could improve the electrophysiological substrate by inhibiting cardiac remodelling and reducing the dispersion of action potential duration, which was beneficial to eliminate reentrants.^30^ Additionally, SSYX can regulate cardiac autonomic nerve function by inhibiting the neural remodelling, thus playing an anti-arrhythmic role.^31^

Our data imply that the use of SSYX post-ablation is an effective and safe treatment strategy for reducing AF recurrence and should be considered following ablation, particularly for patients with persistent AF. Combining SSYX and ablation might be applicable in clinical practice. Maintenance of sinus rhythm after catheter ablation is associated with a reduction in cardiovascular mortality in patients with AF. However, due to the side effects, long-term use of AADs post-ablation cannot be tolerated in many patients. Discontinuation of AADs after the blanking period was generally recommended in the guidelines for the management of AF.^32,33^ This trial showed that long-term use of SSYX post-ablation not only effectively reduced AF recurrence and improved QoL, but also exhibited excellent safety, which may lead to further optimization of management of persistent AF patients.

### Limitations

There are several important limitations in this trial. First, although patients received periodical assessment with planned 24-h Holter monitoring, and otherwise symptom-driven cardiac rhythm monitoring in this trial, there may have been substantial asymptomatic recurrences during follow-up. Using longer or continuous monitoring systems, such as implantable loop recorders or increasing the frequency of 24-h Holter monitoring or 7-day Holter recording, could effectively monitor cases of asymptomatic AF recurrence. The use of patient-driven reporting in this trial for monitoring the recurrence of AF may also contribute to an underestimation of the recurrence rates, as this method can vary in accuracy due to subjective factors and individual inconsistencies. Second, the majority of secondary endpoints in this trial showed no significant differences, primarily due to varied assessment methods and quality across sub-centres, and the lack of a centralized evaluation approach to standardize the acquisition of secondary endpoints. Third, the proportion of participants who did not complete follow-up in the SSYX and placebo groups was 13.3% (61/460) and 15.2% (70/460), respectively, with no significant difference between groups. The balanced proportions of participants who did not complete follow-up in both groups had little impact on the endpoint analysis. Fourth, this trial indicates that SSYX could reduce the recurrence rate of persistent AF in patients following ablation. However, due to the limited sample size, it is currently unclear which specific patient subgroups are most likely to benefit from SSYX treatment. Fifth, this trial utilized the classic SF-36 QoL scoring tool to evaluate patient QoL, without including other symptom assessment tools such as the EHRA symptom classification. Finally, our trial included only Chinese patients, and physicians outside of China did not have much experience in using SSYX. The conclusion of this trial still needed to be confirmed in future international multi-centre trials and in other ethnic groups.

## Conclusions

Treatment with SSYX following radiofrequency catheter ablation for persistent AF reduced the incidence of recurrent atrial tachyarrhythmias and led to clinically significant improvements in QoL during a 12-month follow-up in a Chinese population. Therefore, oral SSYX following catheter ablation is effective and safe in reducing the risk of AF recurrence in patients with persistent AF, which may provide an innovative management strategy for patients with persistent AF after catheter ablation.

## Supplementary Material

ehae532_Supplementary_Data

## Declarations

### Data Availability

The data that support the findings of this trial are available from the corresponding author upon reasonable request.

### Pre-registered Clinical Trial Number

The pre-registered clinical trial number is ChiCTR1900026912.
