# Supplementary material for: Atrial tachyarrhythmia prevention by Shensong Yangxin after catheter ablation for persistent atrial fibrillation: the SS-AFRF trial
Source: Eur Heart J. 2024 Aug 23;45(40):4305–14. doi: 10.1093/eurheartj/ehae532 (PMC11491151; doi:10.1093/eurheartj/ehae532)
Supplement: ehae532_Supplementary_Data [file ehae532_supplementary_data.zip › Revised Supplementary tables.docx]

**Supplementary tables**

**Supplementary table S1 Class I or III antiarrhythmic drugs prescribed post-ablation**

|  | Amiodarone | | Dronedarone | | Propafenone | | Ibutilide | | Mexiletine | | Nifekalant | |
| --- | --- | --- | --- | --- | --- | --- | --- | --- | --- | --- | --- | --- |
|  | SSYX  (n=443) | Placebo  (n=439) | SSYX  (n=443) | Placebo  (n=439) | SSYX  (n=443) | Placebo  (n=439) | SSYX  (n=443) | Placebo  (n=439) | SSYX  (n=443) | Placebo  (n=439) | SSYX  (n=443) | Placebo  (n=439) |
| 0M-3M,  n (%) | 336 (75.9) | 335 (76.3) | 41 (9.3) | 42 (9.6) | 32 (7.2) | 30 (6.8) | 19 (4.3) | 16 (3.6) | 1 (0.2) | 0 | 0 | 1 (0.2) |
| 3M-6M,  n (%) | 113 (25.5) | 122 (27.8) | 18 (4.1) | 24 (5.5) | 3 (0.7) | 3 (0.7) | 1 (0.2) | 0 | 0 | 0 | 0 | 0 |
| 6M-9M,  n (%) | 11 (2.5) | 13 (3.0) | 3 (0.7) | 3 (0.7) | 1 (0.2) | 5 (1.1) | 0 | 0 | 0 | 1 (0.2) | 0 | 0 |
| 9M-12M,  n (%) | 5 (1.1) | 2 (0.5) | 1 (0.2) | 2 (0.5) | 3 (0.7) | 2 (0.5) | 0 | 2 (0.5) | 0 | 0 | 0 | 0 |

**Supplementary table S2. Main Secondary Outcomes**

|  | **SSYX** | **Placebo** | **Estimated Difference**  **(95% CI) *** | ***P* value** |
| --- | --- | --- | --- | --- |
| AF burden (%), Mean (SD) |  |  |  |  |
| 3 months | 2.8 (15.8) | 7.6 (25.7) | -4.8 (-7.9, -1.7) | 0.002 |
| 6 months | 3.3 (17.5) | 7.7 (25.5) | -4.4 (-7.7, -1.1) | 0.025 |
| 12 months | 5.1 (21.0) | 7.9 (26.5) | -2.8 (-6.3, 0.7) | 0.104 |
| Echocardiographic parameters, Median (IQR)* |  |  |  |  |
| LAD (mm) | 38 (34, 41) | 37 (34, 40) | 0.0 (-1.0, 0.0) | 0.324 |
| LVEF (%) | 62 (59, 66) | 63 (59, 67) | -1.0 (-1.0, 0.0) | 0.339 |
| LVEDD (mm) | 48 (45, 51) | 48 (45, 50) | 0.0 (-0.2, 1.0) | 0.094 |
| Time to first AF recurrence, days, Mean (SD) | 172 (95.5) | 147 (76.7) | 25 (-2.0, 51.7) | 0.126 |
| Electrical cardioversion, n (%) | 6 (1.4) | 14 (3.4) | -2.0 (-4.1, 0.2) | 0.060 |
| Stroke, n (%) | 1 (0.2) | 3 (0.7) | -0.5 (-1.8, 0.7) | 0.366 |

Abbreviations: SSYX, Shensong Yangxin; AF, atrial fibrillation; LAD, left atrial diameter; LVEF, left ventricular ejection fraction; LVEDD, left ventricular end-diastolic diameter. IQR, interquartile range.

***** Between-group differences are expressed in a pseudo-median difference calculated with the use of the Hodges-Lehmann estimate based on the Mann-Whitney U test; Because of the use of the Hodges-Lehmann estimator, the estimated difference is not the crude difference between the medians.

**Supplementary table S3. Serum liver and kidney function, and lipids levels.**

|  | **Baseline** | | | | **3 months** | | | **6 months** | | | **12 months** | | |
| --- | --- | --- | --- | --- | --- | --- | --- | --- | --- | --- | --- | --- | --- |
|  | SSYX | | Placebo | *P-*value | SSYX | Placebo | *P-*value | SSYX | Placebo | *P-*value | SSYX | Placebo | *P-*value |
| ALT (n, %) | | 97.3 | 96.4 | 0.522 | 98.4 | 98.0 | 0.769 | 99.0 | 97.8 | 0.330 | 98.3 | 99.0 | 0.722 |
| AST (n, %) | | 97.8 | 97.8 | 0.948 | 99.4 | 98.0 | 0.172 | 98.3 | 99.3 | 0.450 | 99.0 | 99.3 | >0.999 |
| Urea (n, %) | | 100.0 | 99.4 | 0.500 | 99.7 | 100.0 | 0.496 | 99.7 | 100.0 | >0.999 | 100.0 | 100.0 | - |
| Cr (n, %) | | 99.0 | 99.0 | >0.999 | 99.1 | 97.8 | 0.340 | 99.3 | 99.3 | >0.999 | 99.3 | 99.7 | 0.624 |
| TC (n, %) | | 95.7 | 95.9 | >0.999 | 92.4 | 93.4 | 0.734 | 90.1 | 94.8 | 0.060 | 95.6 | 95.2 | 0.835 |
| LDL-c (n, %) | | 93.2 | 94.9 | 0.546 | 93.5 | 94.0 | >0.999 | 93.6 | 94.9 | 0.676 | 97.1 | 96.0 | 0.607 |

Abbreviations: SSYX, Shensong Yangxin; ALT, alanine transaminase; AST, aspartate aminotransferase; Cr, creatinine; TC, total cholesterol;

LDL-c, low-density lipoprotein cholesterol. n, percentage of normal value.
