# Supplementary material for: Atrial tachyarrhythmia prevention by Shensong Yangxin after catheter ablation for persistent atrial fibrillation: the SS-AFRF trial
Source: Eur Heart J. 2024 Aug 23;45(40):4305–14. doi: 10.1093/eurheartj/ehae532 (PMC11491151; doi:10.1093/eurheartj/ehae532)
Supplement: ehae532_Supplementary_Data [file ehae532_supplementary_data.zip › Supplementary statistical analysis_PL.docx]

| **Statistical Analysis Plan (SAP)** |
| --- |
| \| **Statistical analysis plan version** \| **Cause of changes** \| \| --- \| --- \| \| Statistical analysis plan V0.1 \| First release \| \| Statistical analysis plan V0.2 \| Review of SAP before database lock \| |

**December 18, 2022**

Contents

[1. Generalities 3](#_Toc152800245)

[2. Trial overview 3](#_Toc152800246)

[2.1. Research title 3](#_Toc152800247)

[2.2. Objective of the study 3](#_Toc152800248)

[2.3. Study Design 3](#_Toc152800249)

[2.4. Study Population 4](#_Toc152800250)

[2.4.1. Inclusion Criteria 4](#_Toc152800251)

[2.4.2. Exclusion criteria 4](#_Toc152800252)

[2.5. Sample Size 4](#_Toc152800253)

[2.6. Blind method and randomization 5](#_Toc152800254)

[2.7. Treatment 5](#_Toc152800255)

[3. Endpoints 6](#_Toc152800256)

[3.1. Efficacy endpoints 6](#_Toc152800257)

[3.1.1. Primary efficacy endpoint 6](#_Toc152800258)

[3.1.2. Secondary efficacy endpoints 7](#_Toc152800259)

[3.2. Safety endpoints 7](#_Toc152800260)

[4. Selection of populations 7](#_Toc152800261)

[5. Processing of missing data 8](#_Toc152800262)

[6. Statistical analysis Methods 8](#_Toc152800263)

[6.1. General principles of statistical analysis 8](#_Toc152800264)

[6.2. Enrolment Analysis 9](#_Toc152800265)

[6.3. Baseline and demographic characteristics 9](#_Toc152800266)

[6.4. Analysis of efficacy 9](#_Toc152800267)

[6.5. Analysis of safety 12](#_Toc152800268)

[6.6. Additional analysis 12](#_Toc152800269)

# Generalities

SAP:

Statistical analysis will be performed by independent statisticians from PUCRI.

Statistical analysis plan presented below has been written before database lock.

All modifications made to the analysis plan for the initial strategy will be documented in the analysis report.

Software:

Analyses will be performed using SAS software (version 9.4 or updated version).

Planned analyses:

No interim analysis is planned. Analyses will be performed at the end of the study after database lock.

# Trial overview

## Research title

A multicenter, randomized, double-blind, placebo-controlled clinical study of Shensong Yangxin Capsule on prognosis in patients with atrial fibrillation undergoing radiofrequency ablation.

## Objective of the study

Aim to evaluate the effect of Shensong Yangxin Capsule on patients with atrial fibrillation after radiofrequency ablation, to explore more effective clinical treatment methods for these patients, and to provide high-quality clinical evidence for optimizing clinical rational drug use.

## Study Design

A multicenter, randomized, double-blind, placebo-controlled clinical study was adopted in accordance with international norms to evaluate the effect of Shensong Yangxin Capsule on postoperative prognosis of patients with persistent atrial fibrillation after radiofrequency ablation. All patients who were enrolled according to the Chinese Medical Association's《Atrial fibrillation: According to the guidelines of Current Understanding and Treatment Recommendations 2018》, took Shensong Yangxin Capsule or placebo for 1 year on the basis of conventional treatment. and the recurrence rate of patients with atrial fibrillation within 1 year was defined as the primary research endpoint.

## Study Population

Patients must met all of the inclusion criteria listed below, with the exception of any of the exclusion criteria.

### Inclusion Criteria

(1) Age 18-75 years old;

(2) Diagnosed with persistent atrial fibrillation;

(3) History of atrial fibrillation < 5 years;

(4) Radiofrequency ablation for the first time;

(5) Voluntarily participate in this study and have signed informed consent.

### Exclusion criteria

(1) Valvular atrial fibrillation heart disease;

(2) NYHA cardiac function grade Ⅳ or LVEF<40%, LA diameter >50mm;

(3) Moderate to severe left ventricular hypertrophy (wall thickness >1.5cm);

(4) Patients with acute coronary syndrome or patients requiring stent implantation;

(5) Patients with a history of cardiac surgery and catheter ablation in the past 6 months;

(6) History of atrial fibrillation≥5 years;

(7) Hyperthyroidism;

(8) Patients with atrial fibrillation with cardiomyopathy;

(9) Patients with chronic arrhythmia requiring pacemaker installation;

(10) Patients with known bleeding constitution and left atrial/left auricular thrombus or severe hemostasis and coagulation dysfunction;

(11) Severe hepatic and renal insufficiency [ALT≥3 ULN or eGFR<50ml/min/1.7m^2^];

(12) Patients with neuropsychiatric diseases;

(13) Allergic to the ingredients of the study;

(14) Pregnant or lactating women;

(15) Patients who are participating in other clinical studies;

(16) Patients who are deemed unfit by the investigator to participate in this study.

## Sample Size

Referred to《Catheter ablation vs. antiarrhythmic drug treatment of persistent atrial fibrillation: a multicentre, randomized, controlled trial (SARA study)》(European Heart Journal, 2014 Volume 35, Issue 8, Pages 501-507).

Assumed the 1-year recurrence rate of patients with persistent AF after radiofrequency ablation is 39.8% (the duration of an episode of AF/flutter/atrial tachycardia recorded by ECG≥30s 3 months after ablation is considered as a recurrence of AF). We predicted that the 1-year recurrence rate of atrial fibrillation could be reduced to 30% by adding Shensong Yangxin capsule after radiofrequency ablation. Therefore, we may expect that sample size of minimum 368 patients in each study group will provide the study with 80% power to detect a difference in primary outcome at 5% of significance. Accounting for 20% of lost to follow up patients of the trial, we need to include 460 patients in each group and the total sample size is 920.

## Blind method and randomization

Independent biostatistician of Clinical Research Institute of Peking University used SAS9.4 statistical software package to generate random numbers of study drugs by block randomization method on the computer according to the ratio of study group and control group 1:1. And according to this random number, the study drug (Shensong Yangxin capsule or placebo) was packaged and coded by personnels unrelated to the study.

The Randomized and Investigational Drug Management System (RTSM) was used in this study, and the independent biostatistician will provide the RTSM with a list of drug random numbers. Central random method was used in the study. After completing the baseline assessment, investigators were required to log on to RTSM and provide some information (e.g., subject's date of birth, gender, etc.) before randomizing patients if they met the inclusion criteria. The system will generate patient random number and corresponding drug number in order of enrollment time. Each follow-up visit needs to log in the system to apply for a new drug number. The drug number is different, but the drug group is the same.

The second unblinding method was adopted in this study. After the blind state verification, the data was locked, and the main researcher, biostatistician and data administrator conducted the first blind uncover. The corresponding groups of each random number were marked with the code A and B, so as to carry out statistical analysis on all the data. When the statistical analysis was completed and the statistical report was completed, the second unblinding was carried out and the exact groups of the two groups A and B were announced.

## Treatment

**Teat Group:** conventional treatment+ Shensong Yangxin capsule 4 capsules/times, 3 times/day, orally.

**Control Group:** conventional treatment + Shensong Yangxin capsule placebo 4 capsules/times, 3 times/day, orally.

All the above treatments were performed after radiofrequency ablation, and the course of treatment was 1 year

# Endpoints

Efficacy endpoints

### Primary efficacy endpoint

The primary efficacy endpoint was the recurrence rate of atrial fibrillation. The recurrence of atrial fibrillation after ablation was statistically analysed from the following two perspectives

(1) The first recurrence of atrial fibrillation after the 90-day ablation period was used as the primary efficacy endpoint. Among them, the recurrence rate was defined as the duration of atrial fibrillation/flutter/atrial tachycardia recorded by ECG ≥30s from the end of the 3-month blackout period to the end of follow-up, which was considered as the recurrence of atrial fibrillation. The last postoperative follow-up was used as the follow-up endpoint for this analysis. Recurrence of atrial fibrillation during follow-up after surgery was defined as an event, absence of recurrence of atrial fibrillation was defined as censored, and the censored date was defined at the last date of follow-up. Persistent atrial fibrillation was defined as 24 Holter patients with total atrial fibrillation or 2 routine electrocardiograms with an interval of > 7 days, failure to spontaneously recurve, and no sinus rhythm electrocardiograms during hospitalization.

(2) 1-year recurrence of atrial fibrillation from 90 days after ablation to 1 year after surgery was used as the primary efficacy endpoint. Among them, the 1-year recurrence rate of atrial fibrillation was defined as the duration of atrial fibrillation/flutter/atrial tachycardia recorded by ECG ≥30s from the end of the 3-month blackout period to 1 year after surgery, which was considered as atrial fibrillation recurrence. 1 year after surgery was used as the end point of follow-up for this analysis. Recurrence of atrial fibrillation occurring after 1 year was defined censored, and the censored date was 365 days after surgery. Persistent atrial fibrillation was defined as 24 Holter patients with total atrial fibrillation or 2 routine electrocardiograms with an interval of >7 days, unable to spontaneously recurve, and no sinus rhythm electrocardiograms during hospitalization.

With reference to most literatures, the current assessment of recurrence of atrial fibrillation after ablation is mainly based on Holter electrocardiogram (ECG), remote electrocardiogram or the patient's symptoms. In this study, 24h-holter electrocardiogram and electrocardiogram will be used as monitoring means to evaluate recurrence of atrial fibrillation after radiofrequency ablation based on each visit of the patient at the follow-up point and the patient's symptoms.

### Secondary efficacy endpoints

(1) Changes in atrial fibrillation load at 3 months, 6 months, and 1 year after treatment. Atrial fibrillation load was defined as the longest duration of atrial fibrillation episodes, the number of episodes of atrial fibrillation, and the percentage of time patients were in atrial fibrillation during a given monitoring period.

(2) Time of first occurrence of atrial flutter/atrial fibrillation.

(3) Incidence of electrical cardioversion within 1 year after treatment.

(4) Changes of transthoracic echocardiographic indexes at 1 year after treatment.

(5) Incidence of stroke and thromboembolism at 6 months and 1 year after treatment.

(6) Health Survey Summary Form (SF-36) score within 1 year after treatment.

Safety endpoints

(1) Adverse events (AEs): Any adverse medical events occurring between the time subjects signed the informed consent and were enrolled in the study and the last follow -up, regardless of whether there was a causal relationship with the study drug, was considered an adverse event. The sum of "definitely relevant", "possibly relevant" and "unascertainable" associated with the study drug was the adverse reactions (ADRs) of the study drug, and the incidence of adverse reaction was calculated accordingly.

(2) Laboratory indicators: blood routine, urine routine, stool routine, biochemical examination and coagulation function examination;

(3) Clinical physical examination and vitals.

# Selection of populations

All analyses were performed on patients undergoing radiofrequency ablation and will not include patients who were followed for less than 3 months and did not complete the blanking period.

The primary analysis will be for a modified intent to treat population set (mITT), which included all randomized patient with the exclusion of patients that did not provided consent, failure to receive treatment or without any valid data collected after randomization. Following the intention-to-treat principle, patients will be analysed according to the procedure assigned.

Per-Protocol set (PPS) population: a subset of the mITT analysis set, including all patients randomized, treated without major protocol violations/deviation.

pre-defined major protocol violations/deviations are:

- poor compliance in treatment, that is, less than 80% adherence to taking the drug;
- missing data for the primary efficacy endpoint
- major violation of the research protocol, such as non-respect of the randomized procedure allocation and/or duration (wrong procedure followed, premature discontinuation of procedure)

Not pre-defined major protocol deviations identified during a blinded data review before final database lock will be classified.

Safety set (SS): All patients who received at least one treatment after randomization. Patients will be analysed according to procedure received

Measures of efficacy were analysed using mITT and PPS populations, respectively. Safety was analysed by SS population.

# Processing of missing data

In this study, survival analysis was used to analyse the 1-year recurrence of atrial fibrillation for the primary efficacy endpoint. mITT and PPS were both included for the patients who failed to complete the 1-year follow-up due to half-way drop out or missing follow-up. The endpoint was defined as censored in the statistical analysis, and the censored date was the last follow-up date. No carry-over of missing data or multiple fillings of missing data were performed.

Missing data of secondary endpoints and safety endpoints, no filling or other processing is carried out.

Handling of dates: When only part of the date is known, the following rules are used to calculate the duration. If the start time (such as the date of first onset of AF or the date of diagnosis) is partially missing and only the day is missing, then fill in the first day of the corresponding month. If the end date is partially missing and only the day is missing, replace it with the last day of the month. If both the day and month of the date are missing, this date is treated as missing.

# Statistical analysis Methods

General principles of statistical analysis

Statisticians are responsible for developing a statistical analysis plan (SAP) with the principal investigator after the protocol is confirmed. Statistical analysis will be performed with SAS 9.4 (or higher version; SAS Institute, Cary, NC). The sample size is calculated using PASS13 software (NCSS, Kaysville, UT).

A 2-tailed P<0.05 will be considered statistically significant in all statistical tests. (If no other specification). No adjustments for multiple comparisons were made.

Qualitative data will be described with frequencies and percentages; quantitative data will be described with mean and standard error or with median and interquartile interval, minimum and maximum.

Continuous variables will be summarized with mean and SD and compared using paired t-test or Wilcoxon rank test, as appropriate. Categorical variables will be summarized with frequencies and percentages and compared using chi-square test or Fisher exact test, as appropriate. Ranked variables will be compared using Wilcoxon rank test or CMH test.

Enrolment Analysis

Enrollment analysis will summarize the number of enrollment and the number of subjects who completed the trial of each site and lists dropout subjects.

The dataset size between groups, subjects' disposition between sites, dropout rate and termination reasons will be listed in detail

Baseline and demographic characteristics

Baseline characteristics of patients will be described overall and per group.

Demographic and baseline characteristics (age, gender, height, BMI, vital signs, etc.), medical history, family history of atrial fibrillation, drug allergy, etc. of the subjects will be summarized. T-test or Wilcoxon rank-sum test will be used in baseline comparison of continuous variables such as age, height, and BMI between the two groups. The chi-square test or Fisher’s exact test will be used in the baseline comparison of categorical variables such as gender, and medical history

Analysis of efficacy

Both mITT and PPS were used for effectiveness analysis. Analysis performed on PP populations using the same methods can be considered as a sensitivity analysis.

(1) Baseline: The baseline of each relevant efficacy endpoints was described. For comparison methods between groups, refer to the general statistical method above.

(2) Primary efficacy endpoint: The recurrence rate of atrial fibrillation after ablation was statistically described in the two groups respectively.

Survival analysis was used to statistically analyse the recurrence rate of atrial fibrillation after ablation in the two groups. Kaplan-Meier curve of recurrence of atrial fibrillation after ablation was drawn between the two groups, and Log-rank test was used to compare the endpoint between the two groups. Besides, Cox proportional hazard regression model was used to calculate the risk ratio (HR) and 95% confidence interval (CI) of atrial fibrillation recurrence after ablation in the experimental group and the control group to estimate the efficacy, while considering each site as random effect included in the model.

- If atrial fibrillation recurred within 1 year, it was defined as the occurrence of end event. Time to recurrence of atrial fibrillation within 1 year=date of recurrence of atrial fibrillation-(date of radiofrequency ablation+90 days) +1, calculated by day.
- If the visit specified in the protocol was not completed within 1 year, the follow-up could not be completed by the end of 1 year due to loss of follow-up or withdrawal or other reasons, it was defined as censor, censor time=date of last follow-up-(date of radiofrequency ablation +90 days) +1, calculated in days.
- If the visit was completed within 1 year and no recurrence of atrial fibrillation occurred, it was defined as censor, and the censor time in analysis (1) was the date of last visit- (date of radiofrequency ablation+90 days) +1; The censor time in analysis (2) was 365 days.
- If the visits required by the protocol were completed within 1 year and AF recurred after 1 year, the endpoint was defined as event in analysis (1) and the recurrence time was the date of AF recurrence -(date of radiofrequency ablation+90 days) +1; the endpoint was defined as censor in analysis (2) and the censor time was the last visit- (date of radiofrequency ablation+90 days) +1.

(3) Secondary efficacy endpoints

Aimed at changes in atrial fibrillation load at 3 months, 6 months and 1 year after treatment, the mean, standard deviation, median, minimum, maximum, lower quartile (Q1) and upper quartile (Q3) of atrial fibrillation load at each visit site were calculated according to the form of quantitative endpoints. T-test or Wilcoxon rank sum test were used to compare it between the two groups. At the same time, the changes of atrial fibrillation load compared with baseline at 3 months, 6 months and 1 year after treatment were statistically described, and T-test or Wilcoxon rank sum test was used to compare the changes between the two groups.

The time of the first occurrence of atrial fibrillation/atrial fibrillation was analysed as the secondary endpoint. The occurrence days of atrial fibrillation/atrial fibrillation were calculated by the date of radiofrequency ablation surgery as the starting date, the occurrence days= the date of the first occurrence of atrial fibrillation/atrial fibrillation-the date of radiofrequency ablation surgery +1. For patients with atrial flutter/atrial fibrillation, the mean, standard deviation, median, minimum, maximum, lower quartile (Q1) and upper quartile (Q3) of occurrence time were calculated according to the form of quantitative variables. T-test or Wilcoxon rank sum test were used to compare the occurrence time between the two groups.

The secondary efficacy endpoint of transthoracic echocardiography one year after treatment were analysed. The indexes of transthoracic echocardiography mainly included left ventricular end-diastolic volume, left ventricular end-systolic volume, ejection fraction, stroke volume, cardiac output and other evaluation indexes. The mean, standard deviation, median, minimum, maximum, lower quartile (Q1) and upper quartile (Q3) of the results of the evaluation indicators were calculated according to the form of quantitative variables, and T-test or Wilcoxon rank sum test were used to compare the evaluation variables between the two groups.

The incidence of cardiac cardioversion within 1 year after treatment and the incidence of stroke and thromboembolism at 6 months and 1 year after treatment were analysed as secondary efficacy endpoints. The incidence and incidence difference of the two groups in the experimental group and the control group were described according to general principles. Meanwhile, Chi-square test or Fisher exact probability method were used to compare the incidence between the two groups.

SF-36 score within 1 year after treatment were analysed, and the mean, standard deviation, median, minimum, maximum, lower quartile (Q1) and upper quartile (Q3) of each dimension score were calculated according to the form of quantitative endpoints. And T-test or Wilcoxon rank sum test were used to compare the scores of each dimension between the two groups. It includes 8 dimensions and an overall score. The score of each dimension is calculated as follows:

**Table 1: Summary Form of Health Status Survey (SF-36) scoring criteria**

| **8 dimensions + overall** | **Items** | **Scoring methods for each dimension** |
| --- | --- | --- |
| 1. Physiological Function(PF) | 3.1-3.10 | PF=[(actual score - 10) /20] x 100 |
| 2. Physiological Function(RP) | 4.1-4.4 | RP=[(actual score -4) /4] x 100 |
| 3. Body pain (BP) | 7,8 | BP=[(actual score -2) /9] x 100 |
| 4. Overall Health (GH) | 1, 11.1- 11.4 | GH=[(actual score -5) /20] x 100 |
| 5 Vitality (VT) | 9.1, 9.5, 9.7, 9.9 | VT=[(actual score -4) /20] x 100 |
| 6. Social Function (SF) | 6,10 | SF=[(actual score -2) /9] x 100 |
| 7. Emotional Function(RE) | 5.1-5.3 | RE=[(actual score -3) /3] x 100 |
| 8. Mental Health(MH) | 9.2, 9.3, 9.4, 9.6, 9.8 | MH=[(actual score -5) /25] x 100 |
| Overall: Health Change(HT) | 2 | HT=[(actual score - 1) /4] x 100 |

* Total Body Health measures (PCS) include: PF, RP, BP, GH;

* Total Measure of mental Health (MCS) includes: VT, SF, RE, MH.

* Scoring method for each dimension = (actual score - lowest score)/(highest score - lowest score)×100

* Actual score (for each dimension) = Add the scores for each item

Analysis of safety

Safety evaluation is analyzed on SS populations.

Proportion of antiarrhythmic drugs using within 90 days after radiofrequency ablation will be described in each group using frequencies and percentages. The range of antiarrhythmic drugs within 90 days after radiofrequency ablation was defined as the start date of ATC3 drugs between the day of radiofrequency ablation and the 90^th^ day of surgery.

Proportion of adverse events, severe adverse events, adverse reactions, and severe adverse events will be described in each group using frequencies and percentages. If necessary, changes of the laboratory and electrocardiogram variables will be analyzed before and after treatment.

Additional analysis

Additional analyses will be performed to compare primary and secondary endpoints between randomization groups after excluding patients who were still using antiarrhythmic drugs after 90days after radiofrequency ablation. Analysis methods for primary and secondary endpoints analysis described previously will be performed.
